# The demise of calcium-based phosphate binders—is this appropriate for children?

**DOI:** 10.1007/s00467-014-3017-y

**Published:** 2014-12-28

**Authors:** Lesley Rees, Rukshana Shroff

**Affiliations:** Paediatric Nephrology, Great Ormond Street Hospital for Children, Great Ormond St, London, WC1N 3JH UK

**Keywords:** Chronic kidney disease, Children, Mineral bone disorder, Calcium balance, Phosphate binders, Vascular calcification, Bone mineral density

## Abstract

In children with chronic kidney disease (CKD) optimal control of mineral and bone disorder (MBD) is essential not only for the prevention of debilitating skeletal complications and for achieving adequate growth, but also for preserving long-term cardiovascular health. The growing skeleton is particularly vulnerable to the effects of CKD, and bone pain, fractures and deformities are common in children on dialysis. Defective bone mineralisation has been linked with ectopic calcification, which in turn leads to significant morbidity and mortality. Despite national and international guidelines for the management of CKD-MBD, the management of mineral dysregulation in CKD can be extremely challenging, and a significant proportion of patients have calcium, phosphate or parathyroid hormone levels outside the normal ranges. Clinical and experimental studies have shown that, in the setting of CKD, low serum calcium levels are associated with poor bone mineralisation, whereas high serum calcium levels can lead to arterial calcification, even in children. The role of calcium in CKD-MBD is the focus of this review.

## Introduction

Hyperphosphataemia is prevalent in chronic kidney disease (CKD), and particularly so in patients on dialysis. Phosphate retention causes hyperparathyroidism and bone disease, and is also a potent vascular toxin in its own right, as well as through its effects on parathyroid hormone (PTH) and fibroblast growth factor 23 (FGF23) [1, 2]. Phosphate control begins with dietary restriction, but as CKD becomes more severe, this is rarely adequate and phosphate binders become necessary. Unfortunately, compliance with phosphate binders is poor [3] due to their side effects and to the repetitive monotony of the need for their ingestion with every meal and snack.

Phosphate binders are categorised according to whether they are calcium based or calcium-free. There is an ongoing market for non-calcium-containing binders, with new ones currently on trial. The driving force behind this search is (1) the recognition that phosphate control is difficult and remains poor and (2) the relationship between gastrointestinal absorption and retention of calcium excess, and vascular calcification that has been demonstrated in adults. However, only calcium carbonate (CaCO_3_) and acetate are licensed in children, along with sevelamer hydrochloride for those aged >12 years.

The growing skeleton is particularly vulnerable to the effects of CKD-mineral and bone disorder (MBD) as calcium accrual in the skeleton continues from birth until peak bone mass is reached at approximately 30 years of age [4]. However, high calcium levels can lead to arterial calcification, even in children [5, 6]. In adults receiving dialysis, randomised trials have shown that the progression of calcification is greater with calcium acetate than with the calcium-free binder sevelamer [7–9]. These studies have led to recommendations to restrict the use of calcium-based phosphate binders. Importantly, the average age of patients in these studies was 65 years, with many being post-menopausal. While data from adult CKD studies should not be extrapolated to paediatric practice, many paediatricians are increasingly using calcium-free phosphate binders in children. In this review we discuss the role of calcium in CKD-MBD, including calcium homeostasis and the relative merits of different phosphate binders.

## How good are paediatric nephrologists at controlling calcium and phosphate levels in their patients?

Despite national and international guidelines for the management of CKD, many patients have CKD-MBD [10–13]. This is exemplified by data from the International Pediatric Peritoneal Dialysis Network (IPPN) in 153 children worldwide, in whom the PTH varied from country to country, but overall was over fivefold the upper limit of normal in approximately 50 % of patients. The highest levels were associated with high phosphate and lower calcium levels [14]. This level of PTH is associated with symptomatic bone disease: of nearly 900 children in the IPPN, 1.5 % have active bone pain, 5 % limb deformities, 10 % radiological osteodystropy and 5 % osteopaenia [3]. The latest IPPN data shows that for all ages, 14 % of patients have a serum calcium level below the normal range, 24 % have a serum phosphate level above the normal range, 47 % have a serum PTH level above the normal range; in addition, although 21 % are on sevelamer, 22 % are on a calcium supplement other than a calcium-containing phosphate binder [15]. In a cohort of 249 young Dutch adults with onset of end-stage kidney disease (ESKD) before the age of 14 years, 61 % had severe growth retardation, 37 % had bone disease (defined by at least one of the following conditions: deforming bone abnormalities, chronic pain related to the skeletal system, disabling bone abnormalities, aseptic bone necrosis and atraumatic fractures) and 18 % had disabilities resulting from bone impairment [16].

## Regulators of serum calcium in health and in CKD

**Fig. 1:**
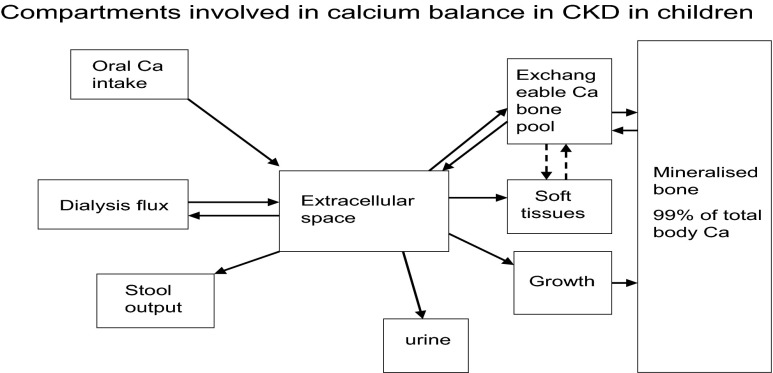
Compartments involved in calcium balance in chronic kidney disease (CKD) in children.* Ca* Calcium. Adapted for children from Moe [17] (used with permission)

**Fig. 2:**
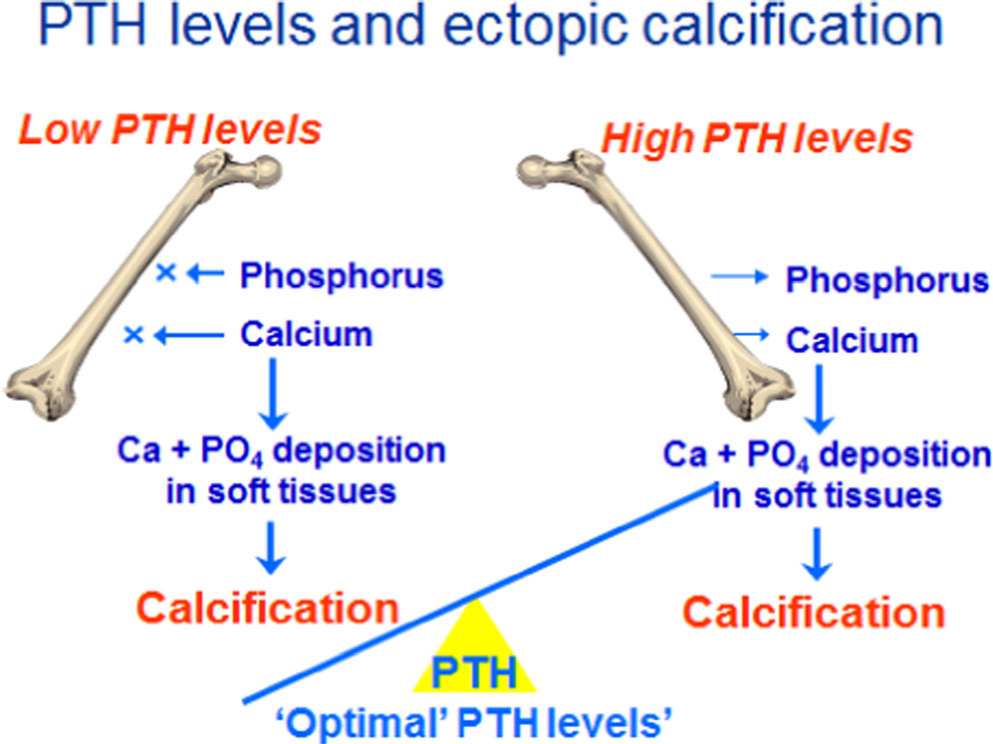
Levels of parathyroid hormone (*PTH*) and ectopic calcification.* PO*_*4*_ Phosphate

**Fig. 3:**
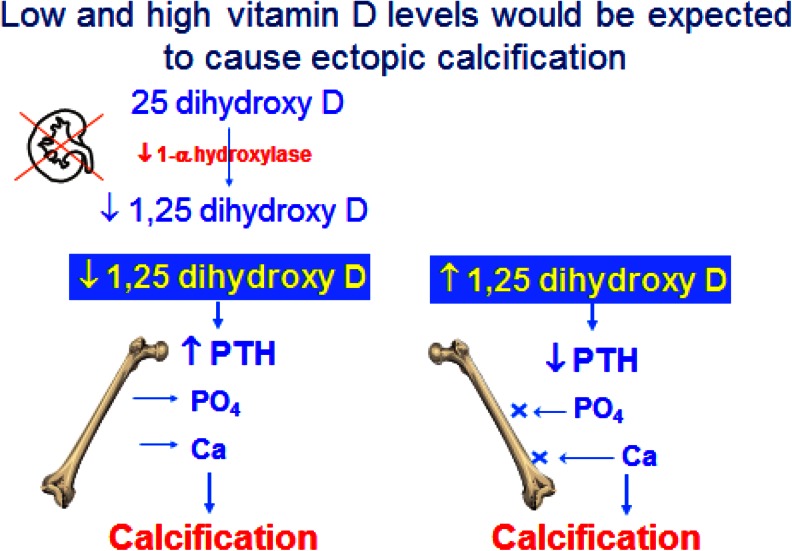
Low and high vitamin D levels would be expected to cause ectopic calcificationᅟ

Hypercalcaemia is not just a result of high calcium intake. Both low and high blood PTH levels and 1,25(OH)D (1,25 hydroxy-vitamin D) can cause hypercalcaemia. Under normal circumstances, after calcium has been absorbed into the blood it can be exchanged as part of bone remodelling, incorporated into bone for growth or excreted in the urine (Fig. 1) [17]. The plasma calcium therefore does not indicate the total body calcium, but is a reflection of calcium movement into and out of the extracellular fluid (ECF). Indeed, just as hypercalcaemia does not necessarily mean that total body calcium is in excess, normocalcaemia does not mean that it is adequate. The ionised calcium in the ECF is crucial for the function of many enzyme systems throughout the body and, in particular, signalling pathways. Its maintenance within a strict, narrow range is therefore essential. A fall in plasma ionised calcium level activates calcium sensing receptors in the parathyroid gland which, in turn, activate PTH production. All of the actions of PTH are to increase the plasma calcium level, which it does by increasing renal tubular calcium reabsorption, increasing production of 1,25(OH)_2_D, which in turn increases absorption of calcium from the gut, and by causing an efflux of calcium from bone. Therefore, if calcium influx from the gut is low, PTH stimulation will result in calcium being taken from bone, and if levels rise in the blood, PTH is suppressed and renal tubular reabsorption of calcium is decreased so that calcium is excreted in the urine. Prolonged, uncontrolled PTH secretion will result in downregulation of PTH receptors so that a higher plasma calcium level is needed to suppress PTH, resulting in hypercalcaemia. On the other hand, low PTH levels will result in adynamic bone that is unable to buffer influxes of plasma calcium, also resulting in hypercalaemia (Fig. 2). This bimodal effect is also seen with vitamin D (Fig. 3) [18]. High 1,25(OH)_2_D (1,25 dihydroxy-vitamin D; calcitriol) levels stimulate calcium absorption and suppress PTH, whereas low levels decrease calcium absorption and increase PTH, and are also associated with higher C-reactive protein levels.

**Fig. 4:**
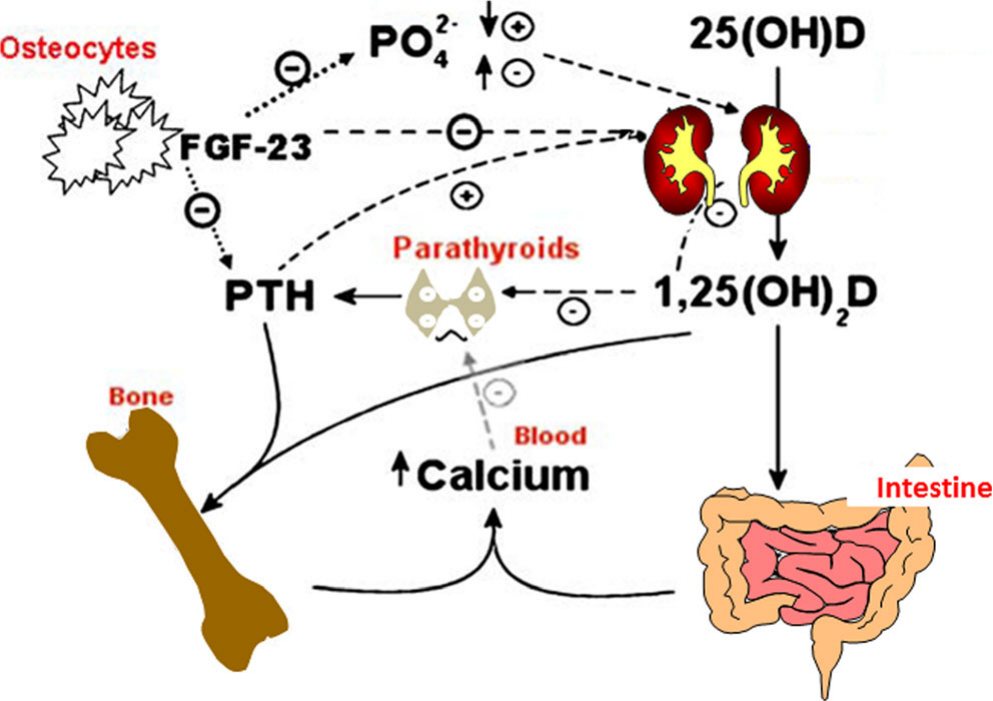
Mechanisms involved in calcium homeostasis.* FGF-23* Fibroblast growth factor, *PTH* Parathyroid hormone

Recent studies have also suggested that FGF23, a bone-derived protein that was first believed to be mainly involved in phosphate homeostasis, is also involved in calcium regulation. FGF23 increases renal phosphate excretion by downregulating the sodium–phosphate co-transporter in the proximal tubules, thereby, at least in early CKD, increasing phosphorus (P) excretion [19]. It also suppresses 1α-hydroxylase and increases 24-hydroxylase activity, so that there is a reduced production and increased degradation of 1,25(OH)_2_D [19]. Recent work has shown that there may be a threshold effect of serum calcium on FGF23 regulation, such that FGF23 is not stimulated when the calcium level is very low (essentially because this would reduce calcitriol and further aggravate hypocalcaemia). Studies in uraemic rats support this finding: in the presence of hypocalcaemia FGF23 production was not increased by low calcitriol or high PTH levels [20]. Interestingly, sevelamer, but not Ca-based binders, have been shown to decrease FGF23 levels in predialysis CKD patients, but this effect could not be attributable to improved phosphate or 1,25 (OH)_2_D levels [21], suggesting that a relative hypocalcaemia in these subjects did not allow for FGF23 production irrespective of phosphate or 1,25 (OH)_2_D levels. A study in haemodialysis patients has shown that the use of higher dialysate Ca concentrations, as well as the administration of calcitriol and a calcium-based phosphate binder were associated with higher final serum FGF23 levels [22]. In a previous study, we found that in a population of children with well-controlled serum phosphate, high FGF23 levels were seen with a progressive decline in estimated glomerular filtration rate (eGFR) and showed a positive correlation with serum calcium [23]. The complex interactions of calcium, phosphate, vitamin D and FGF23 are shown in Fig. 4.

## The need for calcium in growing bones

The growing skeleton has a high demand for calcium, whereas adults may have ‘adynamic’ bones that are unable to incorporate calcium [24]. Clinical measures of bone mineral density (BMD) and bone biopsies have shown defective mineralisation as early as CKD stage 2 [25], and the prevalence increases to 90 % in patients on dialysis [26]. In dialysed children, the mineralisation defect was found to persist after treatment with active vitamin D sterols combined with either calcium-based or calcium-free binders and were associated with a concomitant increase in FGF23 levels [27]. Importantly, on bone biopsies in a cohort of 160 children on peritoneal dialysis, the same group showed that low serum calcium and high PTH levels were associated with defective mineralisation, irrespective of bone turnover [28]. This finding has been further corroborated by dual energy x-ray absorptiometry (DEXA) studies: BMD loss was been attributed to low calcium and high PTH levels and was associated with increased fracture risk [29]. In a recent study in 5- to 21-year-olds with CKD stages 2–5, including patients on dialysis, lower calcium levels were associated with baseline and progression of BMD loss; one standard deviation decrease in BMD was associated with a twofold increase in fracture risk. Adynamic bone disease has a high prevalence, being observed in 40–50 % of adult and almost 30 % of paediatric ESKD patients [30–32].

**Fig. 5:**
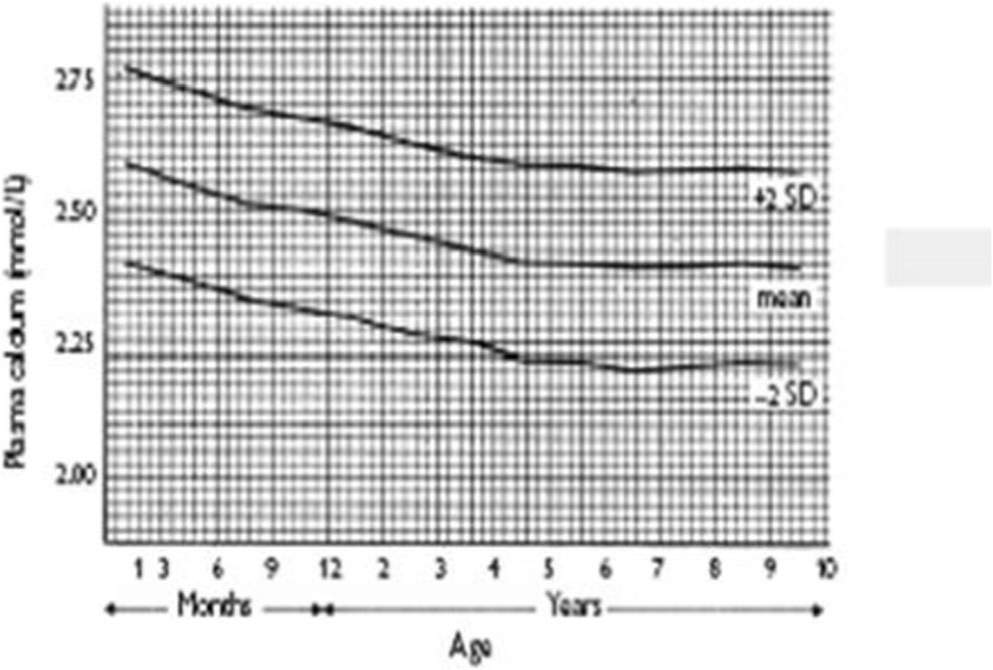
Normal range for calcium throughout childhood.* SD* Standard deviation

One factor that is often forgotten is that the normal range for plasma calcium is highest at birth, falling progressively thereafter, but it does not reach adult values until the age of 4 years. This may not be recognised by clinicians (Fig. 5).

## Clinical studies of the role of calcium in vascular calcification

Converging evidence from clinical and experimental studies suggests a role for high serum calcium levels, particularly when associated with high phosphate levels, in the development and progression of vascular calcification. Changes in arterial structure as well as loss of compliance of arteries have been reported as early as in the second decade of life in children on dialysis [5, 33–39], with approximately 20 % of children on dialysis shown to have coronary artery calcification on computed tomography (CT) scams, increased carotid intima-media thickness (cIMT) and calcified arteries [5, 36, 39]. Evidence of vascular calcification on CT scans is directly related to a high serum calcium × phosphate (Ca–P) product, serum phosphate level, intake of calcium containing phosphate binders and plasma PTH levels [34]. In a postmortem analysis of 120 children with CKD, soft tissue and vascular calcification was associated with the use of active vitamin D and calcium-containing phosphate binders [40]. Progressive increase of cIMT has been associated with higher serum Ca–P product and PTH [5, 41, 42]. In the seminal paper by Goodman et al., not only were the patients with coronary artery calcification older than those without calcification, but they also had a significantly higher mean serum Ca–P level (5.2 vs. 4.5 mmol^2^/L^2^) and almost double the intake of calcium from binders (6,456 vs. 3,325 mg/day) [5]. The serum phosphate, Ca–P and PTH levels were very high in both patient groups, and the calcium intake from phosphate binders was significantly above the K/DOQI (National Kidney Foundation Kidney Disease Outcomes Quality Initiative) recommended limit of 1,500 mg/day, suggesting that these patients were at a substantial risk of ectopic calcification [5]. Moreover, transient increases in calcium that occur in relation to dialysis [43] and vitamin D or binder intake may go unrecorded, but these can impact on ectopic calcification, particularly in the setting of high phosphate conditions. In the ‘Treat-to-Goal’ study, the calcium-treated group had significantly more hypercalcaemic episodes than the sevelamer group [8], and the extent of arterial calcification was directly related to the number of episodes of hypercalcaemia during the preceding 6 months [8].

Studies on the role of calcium intake, from diet, binders or dialysate, on vascular calcification have not been performed in children. However, it is important to remember that the purpose of the calcium homeostatic system is to maintain the ECF calcium levels within a very tight range; consequently, serum calcium levels are a poor, and sometimes misleading, marker of total body calcium level. A recent study has shown that elevated serum calcium, phosphate and Ca–P product levels, but not dietary calcium or phosphate consumption, are associated with increased coronary artery calcification [44].

## Experimental studies of the role of calcium in vascular calcification

Vascular calcification is an active, highly regulated process and not merely a passive deposition of calcium and phosphate in dead or dying cells [45]. In response to raised extracellular calcium and phosphate levels, vascular smooth muscle cells (VSMCs) undergo specific phenotypic changes, including apoptosis, osteo/chondrocytic differentiation and the release of small membrane-bound bodies called vesicles that form a nidus for the deposition of basic Ca–P in the form of hydroxyapatite nanocrystals [6, 45, 46]. VSMC transformation to an osteo/chondrocytic phenotype is characterised by the upregulation of bone-specific transcription factors and matrix proteins, including Runx2/Cbfa1, osterix and alkaline phosphatase, which in turn lead to accelerated calcification [6, 47–50]. Raised serum phosphate level is a key factor that triggers osteoblastic differentiation of the VSMC [49].

Using intact arteries from children, we have shown that calcification in the vessel wall begins in pre-dialysis CKD stages 4 and 5 but is significantly greater in dialysis patients, with the calcium load in the vessel wall increasing linearly with time on dialysis and strongly correlated with the mean time-averaged serum Ca–P product [6]. When these vessel rings were cultured in graded concentrations of calcium and phosphate, normal and pre-dialysis vessels were resistant to calcification, but dialysis vessels showed accelerated calcification in high calcium and phosphate media [51]. The synergistic effect of the Ca–P product in driving calcification may be related to hydroxyapatite nanocrystal formation and their lysosomal degradation, leading to very high intracellular calcium levels and apoptotic cell death [52]. Also, Ca–P nanocrystals upregulate bone proteins, including bone morphogenetic protein 2 (BMP-2) and osteopontin [53, 54]. Calcium plays a key role in inducing apoptosis and in the formation and release of hydroxyapatite-laden matrix vesicles: when VSMCs in vitro are incubated in high calcium conditions, apoptotic cell death releases more calcium, which in turn may drive further apoptosis [55]. Apoptotic bodies form a nidus for calcification, and the propensity of these to calcify is markedly increased after calcium and phosphate treatment [56, 57]. Thus, once a nidus of calcification forms in the extracellular matrix of VSMCs, its uptake and phagocytosis will promote further calcification. Importantly, in the presence of a high phosphate level, even a small increase in calcium in the culture medium significantly increases calcification, and experiments have confirmed that calcium is a key mediator of VSMC damage and calcification [51].

## Calcium balance in health

In health terms, calcium balance is a measure of the calcium ingested and used for bone remodelling, minus the amount removed in urine and lost in stool. In adults who are not deficient in calcium or vitamin D, about 30 % of dietary calcium is absorbed, and the balance would be expected to be neutral [58]. In contrast, children require a positive calcium balance to allow for growth, particularly during periods of rapid bone accumulation, i.e. infancy and puberty.

### Gastrointestinal calcium absorption

Calcium is absorbed mainly in the upper intestine by both passive paracellular diffusion and by vitamin D-dependent active transport. The first step of the active transport process also involves passive diffusion into the enterocytes via the TRPV6 calcium channel. The next step is 1,25(OH)_2_D binding to vitamin D receptors in enterocytes, thereby increasing expression of the calcium transporter protein calbindin and facilitating calcium transport through the cell. The third step is transport into the ECF against a concentration gradient. This step requires energy, in the form of calcium ATPase or sodium/calcium exchange. If calcium intake is normal, the majority of the absorption of calcium is by the active vitamin D-dependent pathway [59].

Intestinal calcium absorption may, therefore, be impaired by low calcium intake and low levels of vitamin D. Passive absorption is dependent on the concentration gradient between the intestinal calcium and the ECF. Intestinal calcium absorption is also reduced by other gut contents which may bind it, such as phytate and oxalate, and by steroids and magnesium deficiency [60]. In healthy children with a low dietary calcium intake, a greater proportion is absorbed (approximately 34 %), and the calcium level is proportional to circulating 25(OH)D levels. In healthy children with higher dietary calcium intakes, vitamin D has less of an influence on calcium absorption, and proportionately less calcium is absorbed (approximately 29 %), i.e. there is adaptation to low 25(OH)D levels and dietary calcium intake [60, 61]. What is not yet known is what proportion of calcium is absorbed at very high calcium intakes.

### Calcium requirements throughout life

The vast majority of the total body calcium (99 %) is in the skeleton, with 0.6 % in soft tissues and 0.1 % in the ECF [10]. There is a progressive increase in total skeletal calcium throughout life, from approximately 25 g at birth to 900 and 1,200 g in adult women and men, respectively. Calcium balance, therefore, needs to be positive throughout childhood. However, the amount of calcium that can be incorporated into the skeleton has a threshold above which any increase in dietary calcium has no further effect. This threshold is influenced by age, such that during periods of rapid growth, i.e. infancy and adolescence, calcium balance is at its most positive. Table 1 illustrates this and clearly shows the very high calcium requirements in the first year of life. Requirements decrease considerably when growth has finished [60, 62].

**Table 1:** Calcium intake above which no further incorporation of calcium into bone occurs and calcium balance per day at different ages [62]

Age (years)	Calcium threshold (mg/day)	Balance per day (mg/day)
0–1	1,090	503 ± 91
2–8	1,390	246 ± 126
9–17	1,480	396 ± 164
18–30	957	114 ± 133

## Calcium balance in CKD

### Diet, calcium and vitamin D

The anorexia of CKD and a diet prescribed to provide a low protein and phosphate intake is very likely to have a low calcium content as well. Furthermore, high-density feeds may be necessary in children on dialysis who need a reduced feed volume with extra protein to allow for losses in the dialysate. The solubility of such feeds may decrease the absorption of minerals [63]. In addition, vitamin D deficiency is present in 60–80 % of children with CKD, even in pre-dialysis CKD children, due to the following possibilities (1) children with CKD may be less active with, consequently, less sunlight exposure; (2) the endogenous synthesis of vitamin D in the skin is known to be reduced in CKD; (3) ingestion of foods that are natural sources of vitamin D may be reduced; (4) proteinuria may be accompanied by high urinary losses of vitamin D binding protein, leading to increased renal losses of all vitamin D metabolites; (5) a low calcium diet can lead to a depletion of vitamin D stores, as the resultant hyperparathyroidism will lead to a rapid conversion of 25 (OH)D to active metabolites [64, 65]. Whether replacement of nutritional vitamin D in CKD can improve calcium absorption is less clear. A randomised controlled trial (RCT) of children with pre-dialysis CKD has shown a delay in the development of hyperparathyroidism in those treated with ergocalciferol l [64]. In contrast, a study of adults on haemodialysis who had very low calcium absorption values at baseline did not show any improvement with cholecalciferol supplementation that raised 25 (OH)D levels to 50 ng/mL. The authors of this study suggest some possible explanations: (1) vitamin D and PTH receptor expression are downregulated by low 1,25(OH)_2_D and/or high PTH so that much higher 1,25(OH)_2_D and PTH concentrations are required to achieve calcium absorption than could be achieved by reasonable cholecalciferol supplementation and/or (2) there is inhibition of calcium absorption because of some condition specific to uraemia (e.g. metabolic acidosis) [66].

True calcium balance studies are difficult to conduct, and very few such studies are reported in the literature. In a 3-week, crossover, calcium balance study of eight adults with CKD 3 and 4 randomised to receive either no calcium carbonate or 1,500 mg/day, patients were in neutral balance during the placebo phase and positive calcium balance during the supplemented phase. There was no benefit to the phosphorus balance. Oral and intravenous ^45^calcium demonstrated net bone balance, but less than overall calcium balance, suggesting that some calcium was deposited in soft tissues [67]. This is the most powerful evidence to date of calcium retention in CKD.

### Sodium bicarbonate, proton pump inhibitors and iron

As well as diet, medications used in children with CKD may affect calcium absorption. For the passive diffusion of calcium from the ileum and jejunum into the ECF, the calcium must be in solution. The first step depends on calcium salt dissolution in the acid pH of the stomach. However, when food contents move into the duodenum, the intestinal mucosa needs protection from the acid and so bicarbonate is secreted. This increases the local gut content of CO_3_^2−^ so that if the concentration of calcium and carbonate exceeds the solubility limit, intestinal precipitation of CaCO_3_ will occur and affect both the phosphate binding capacity of calcium-containing phosphate binders and the absorption of calcium, i.e. calcium will precipitate as CaCO_3_ if the calcium concentration and the CO_3_^2−^ is high [68]. The commonest diagnosis in young children with CKD is CAKUT (congenital anomalies of the kidney and urinary tract). Such children often lose bicarbonate through the renal tubules even on dialysis and need large doses of oral sodium bicarbonate.

Anything that increases the pH in the stomach will therefore affect calcium salt dissolution and decrease calcium absorption. A study of calcium absorption from CaCO_3_ in those with achlorhydria compared to controls showed that the amount of calcium absorbed was higher in the normal subjects due to the need for a low pH to dissolve the tablets [69]. In patients on proton pump inhibitors, calcium absorption has been reported to be as low as 3.5 % [70]. Many children with CKD are treated with such drugs.

Phosphate binders must be given with food and must not be given at the same time as iron preparations as they form insoluble compounds in the gut.

### Body compartments and calcium balance in children on dialysis

In contrast to the healthy state, there are extra dimensions in CKD that affect calcium balance. During dialysis there may be either a net gain from or loss into dialysate, depending on the concentration gradient between the calcium in the blood and that in the dialysate. If influx into the ECF exceeds the body’s capacity to remove it, such as if calcium cannot be excreted in the urine because of reduced urine output (as in some patients with CKD stage 5), or cannot be taken up by the bone exchangeable pool, it has to go somewhere and so may be deposited in the blood vessel wall or in soft tissues. The dynamics of the interchangeable calcium pool on bone that buffers changes in plasma calcium in the blood is incompletely understood, but it seems to be different from the cell-mediated process that is required for growth [17] (Fig. 1).

Intestinal cell shedding leads to an obligatory loss of calcium in stool. This is particularly important in low-birth-weight infants in whom faecal calcium excretion is greater than urinary excretion, at 15.5 ± 8.9 mg/kg/day (7.2 ± 4.1 % of intake), with some studies reporting even higher values [71]. It is not known when this changes to the lower adult values of 2 mg/kg/day.

## Suggested calcium intakes in CKD

In order to try to prevent the deposition in soft tissues of absorbed calcium that might be surplus to need, the K/DOQI nutritional guidelines recommend restricting the upper limit of calcium intake in CKD to <2 × the dietary reference intake, which is less than the upper limit recommended for normal children in those aged less than 8 years and at the upper limit of 2,500 mg for children exceeding that age (Table 2) [72]. The K/DOQI nutritional guidelines also state that ‘the challenge is to ensure that an adequate calcium intake is achieved’, recommending calcium supplementation for infants with CKD, who may not get adequate amounts of calcium if not breast fed, if low-electrolyte infant formulas are required or if fluids are restricted, and for children and adolescents on dialysis in whom a phosphate-restricted diet results in a serious calcium deficit, when typically the dietary calcium intake is <500 mg per day. Calcium-containing phosphate binders may then be the primary source of elemental calcium in the diet.

**Table 2:** Kidney Disease Outcomes Quality Initiative guidelines for calcium restriction in chronic kidney disease [10]

Age	Dietary reference intake (mg)	Upper limit of calcium intake for normal children (mg)	Upper limit of calcium intake in CKD (mg)
0–6 months	210	ND	≤420
7–12 months	270	ND	≤540
1–3 years	500	2500	≤1,000
4–8	800	2500	≤1,600
9–18	1,300	2500	≤2,500

*CKD* Chronic kidney disease, *ND* not defined

The need to restrict calcium intake from binders has to be balanced against the potential for inadequate provision of dietary calcium. K/DOQI does acknowledge, however, that ‘There are no data on calcium retention as a function of increased long-term calcium intake in patients with CKD, and it is impossible to accurately assess the actual absorption of calcium derived from binders, which is in large part dependent upon the kind and amount of food present in the stomach with the binder’ [10]. These calcium intake guidelines have recently been approved by the K/DOQI nutrition group [11].

### How much calcium is absorbed from CaCO_3_ and calcium acetate?

The amount of calcium that is absorbed from a dose of calcium-containing phosphate binder is unknown. It is likely to vary between individuals and according to dietary protein, phosphate, fibre and sodium intake, the vitamin D prescription and when the binder is taken in relationship to food.

CaCO_3_ is most soluble at pH 1–3 and binds to phosphate best at pH >5, with very little binding below that. _._ Short-term studies in adults have shown that for a given dose of elemental calcium, calcium acetate binds twice as much phosphate at the same dose of elemental calcium as CaCO_3_ and, therefore, less calcium is absorbed. One meal balance study showed that the ratio of milligrams phosphate bound to milliequivalents calcium absorbed was 6.8 with calcium acetate and 2.5 for CaCO_3._ However, long-term studies of CaCO_3_ versus calcium acetate have just looked at the difference in the incidence of hypercalcaemia between the two drugs, and this has given variable results [73].

Patients on conventional three-times-a-week haemodialysis or peritoneal dialysis are those who have the most problems with hypercalcaemia due to calcium-containing phosphate binders because of their reduced ability to excrete calcium in the urine. However, the most common cause of CKD in childhood is CAKUT, so many children are not oliguric and continue to produce urine volumes appropriate to their fluid intake; consequently, calcium restriction is less necessary. Use of calcium neutral dialysate (1.25 mmol/L) allows for the prescription of larger doses of calcium.

Calcium absorption is greater if the binder is taken between—rather than with—meals, to prevent it acting as a calcium supplement. Absorption will also vary with plasma 1,25(OH)2D levels, being as low as 3 % in the deficiency state to presumably higher than the expected normal range in patients who are prescribed activated vitamin D, when hypercalcaemia may occur.

## Clinical trials of phosphate binders

The long-term cardiovascular effects of phosphate binder therapy remain controversial. A recent randomised clinical trial in adults with a GFR of 20–45 ml/min/1.73 m^2^ demonstrated an increase in arterial calcification in those patients receiving calcium, lanthanum and sevelamer binder therapy, but not in the placebo-treated patients [74]. Another study showed progressive arterial calcification in predialysis patients on a low-phosphorus diet alone; progression was less in CaCO_3_-treated patients and absent in sevelamer-treated patients [75]. The Independent trial demonstrated improved survival in predialysis patients randomised to sevelamer as compared to those treated with CaCO_3_ [9, 76]. In adult dialysis patients, sevelamer was also found to reduce the progression of coronary artery calcifications, but not to be associated with reduced mortality [77]. However, what has clinched the demise of calcium-based phosphate binders in adults is a recent meta-analysis of 11 RCTs, with 2,312 patients on non-calcium-based phosphate binders and 2,310 patients taking calcium-based phosphate binders. There was a 22 % reduction in all-cause mortality in the non-calcium group. A subanalysis of seven RCTs (704 patients) in which vascular calcification had been measured showed that non-calcium based binders resulted in less progression of vascular calcification [78].

Recent studies in adults have shown that a regimen based on a combination of sevelamer + calcium-based binders was capable of effectively managing hyperphosphataemia without hypercalcaemia at reduced financial burden [79]. The average dose of sevelamer was 2.8 g per day, which is substantially lower than the average daily dose (6.5 g) in the study of Chertow et al. [8]. The combination therapy was better tolerated and showed higher patient compliance than CaCO_3_ or sevelamer monotherapy [80]. In haemodialysis patients this combination therapy averted both the hypercalcaemia of CaCO_3_ and the adverse effects of sevelamer hydrochloride when each was used as monotherapy [81]. Also, provided patients took their prescribed total daily dose of binders, it did not matter if different phosphate binders were taken at the same meal or at separate ones, according to their preference [82].

Only two studies have examined calcium acetate versus sevelamer in children. In an 8-week crossover study in children, sevelamer and calcium acetate were equally effective at reducing serum phosphate levels, but significantly less hypercalcaemia occurred in the sevelamer group [83]. In the second study, bone biopsies suggested that the sevelamer group had reduced bone formation at the 8-month follow-up, but the numbers were too small for comparison [26]. Without evidence, clinical practice is highly variable, with many nephrologists using sevelamer, which is the more expensive option and little is known about its long-term effects in children.

## What new phosphate binders will soon be available for children?

The ideal phosphate binder would be one that has a high affinity for phosphate, is long acting with a small tablet load, has an acceptable taste and texture and is without side effects, is not absorbed, can be administered down a feeding tube and is inexpensive. No phosphate binder that is currently available fulfils all of these criteria. The problem is the increased cost associated with non-calcium-containing binders, which has a substantial effect on prescribing budgets, although this may change when patents expire.

### Colestilan

Colestilan (MCI-196) is an anion exchange resin that absorbs bile acids and phosphorus in the gastrointestinal tract. The absorption of bile acids has a lipid-lowering effect as well, particularly serum low-density lipoprotein cholesterol. In addition to tablets it is available as granules that can either be swallowed or administered via an enteral feeding tube. Trials in adults are complete and are ongoing in children.

### Iron-based binders

The use of iron-based phosphate binders is attractive as there are the potential additional advantages of providing iron and being calcium-free. In a randomised, placebo controlled trial of adult haemodialysis patients, ferric citrate controlled phosphorus compared with placebo with comparable safety profiles. Patients on ferric citrate required less intravenous iron and less erythropoietin-stimulating agent [84]. Trials are due to start in children.

## Conclusion

It cannot be assumed that calcium intake is adequate in all children with CKD, and the amount of calcium absorbed from phosphate binders is unknown. Studies of phosphate binders in adults should not be extrapolated to children: children need a positive calcium balance for healthy bone growth, the spectrum of causal diseases are different and, in particular, urine output is often not impaired even on dialysis.

Careful assessment of calcium intake is required in children with CKD to ensure that calcium intake is adequate but not in excess. Studies are needed to explore calcium absorption from calcium-containing binders.
